# Transcutaneous electrical nerve stimulation for women with primary dysmenorrhea: Study protocol for a randomized controlled clinical trial with economic evaluation

**DOI:** 10.1371/journal.pone.0250111

**Published:** 2021-05-20

**Authors:** Jéssica Cordeiro Rodrigues, Mariana Arias Avila, Patricia Driusso

**Affiliations:** 1 Women’s Health Research Laboratory (LAMU), Physical Therapy Department, Universidade Federal de São Carlos, São Carlos, SP, Brazil; 2 Laboratory of Research on Electrophysical Agents (LAREF), Physical Therapy Department, Universidade Federal de São Carlos, São Carlos, SP, Brazil; University Medical Center Utrecht, NETHERLANDS

## Abstract

Primary dysmenorrhea (PD) is a common gynecological disorder characterized by cramping pain in the lower abdomen during menstruation, in the absence of identifiable pathology. While Transcutaneous Electrical Nerve Stimulation (TENS) is used to promote PD pain relief, there is still a need to understand the parameters and cost-effectiveness of this intervention. As such, this study aims to disclose a study that will evaluate the effectiveness and cost-effectiveness of TENS on pain intensity in women with PD. This is a protocol for an assessor-blinded randomized controlled trial that includes 174 women with PD, >18 years old, with menstrual pain greater than or equal to four points in the Numerical Rating Scale. Participants will be randomized into three groups (active TENS, placebo TENS, and educational booklet). Participants allocated to the active TENS group will receive electrical stimulation (F = 100 Hz, pulse duration = 200 μs, for 30 min). In contrast, the placebo TENS group will receive placebo stimulation (same parameters, but for only 30 s, and then the current will be ramped off over a 15-s time frame) for three consecutive months during menstruation. Participants allocated to the educational booklet group will receive instructions about health promotion and education actions related to PD. A blinded assessor will evaluate the pain intensity (Numerical Rating Scale), presenteeism (Stanford Presenteeism Scale), Health-Related Quality of Life (Short-Form 6 dimensions and SF-36), and health costs of menstrual pain. This is the first study to propose a health economic evaluation while assessing the benefits of using TENS to treat PD symptoms. It is hypothesized that active TENS will be more effective than placebo TENS or the educational booklet in improving clinical outcomes in the short-, medium- and long-term. The study will also provide information about the cost-effectiveness of TENS, which can be used by policy makers to improve PD care in public and private health systems.

## Introduction

Primary dysmenorrhea (PD) is a common gynecological condition characterized by pelvic/abdominal pain before or during the menstrual period in the absence of pelvic disorder [1, 2]. It is believed that the prostaglandin release that occurs after the progesterone levels fall at the end of the luteal phase of the menstrual cycle is responsible for the uterine contractions and pain that characterize this disorder [3]. The prevalence of PD varies from 45% to 95% [4] in women of reproductive age [1]. In Mexico, a survey of 1,266 university students showed a prevalence of 66.9% in women aged between 21 and 23 years [5].

Common symptoms associated with PD are nausea, headache, tiredness, intestinal dysfunction, low back pain, irritability, and adynamia [2]. Often, PD and its symptoms lead to disability in performing daily life activities, being the leading cause of absence from school [6] and work [7] of adolescents and women, respectively. It was estimated that this phenomenon would cost approximately USD 2 billion of economic loss in the mid-1980s [8]. Among the most used interventions for PD are analgesics, nonsteroidal anti-inflammatory drugs, and hormonal contraceptives [9]. Nonetheless, they may present a failure rate of 20–25% and adverse effects such as indigestion, headaches, and drowsiness [10]. Consequently, nonpharmacological alternatives may be used to obtain symptom relief; the most common are regular physical activity [11], topical heat [12], acupuncture [13], and transcutaneous electrical nerve stimulation (TENS) [14–17].

TENS is widely used for any kind of pain [18, 19], and is a non-invasive technique that uses electrical stimulation applied through the skin to produce analgesia by peripheral nerve stimulation [20], among other physiological effects. TENS works by activating the δ-opioid and μ-opioid receptors in both the central and peripheral nervous systems [18, 19]. TENS can lead to increased blood flow to the targeted area by the axonal reflex [21], and, when used during a PD episode, TENS may decrease uterine ischemia as it may increase blood flow in the area of the dermatome corresponding to the uterus [16, 22]. TENS is recommended for women not willing to use conventional therapy for PD, with a recommendation level B [23]. TENS has been widely used for pain relief in PD [15–17, 24, 25], but the lack of information on the parameters used, randomization, and outcomes renders it difficult to replicate such studies [14, 16].

Economic analyses are essential in administrating and managing health services and improving healthcare quality based on scientific evidence [26]. Usually, public policies focused on a disease come from studies that identify the best evidence-based intervention with the lowest costs by analyzing the cost-effectiveness and cost-utility of that intervention. This allows governments to correctly direct finite resources to address the most critical issues in public health problems [27]. With the number of women affected by PD, this condition should be considered a public health problem that must be addressed by policymakers. Although it is known that women with PD are willing to spend more money on a cure for their symptoms compared to women without PD [28], and may spend two- to threefold more on medical consultations [29], the costs associated with PD treatment are still not clear [28], especially regarding nonpharmacological interventions such as TENS.

The general objectives of this research are to investigate if TENS can be effective in treating PD symptoms and to determine the cost-effectiveness associated with this intervention. The hypothesis of the study is that TENS will be effective and cost-effective for the treatment of PD symptoms. This article aims to describe the methods and statistical analysis of this study so that this information can be made public.

## Methods and analysis

### Study design and ethical aspects

This is a study protocol for a randomized controlled clinical trial with a health economic evaluation. The research was approved by the Ethics Research Committee of the Universidade Federal de São Carlos (UFSCar) (CAAE: 16530619.3.0000.5504, protocol number: 3.588.121). This study was registered at the Brazilian Registry of Clinical Trials (ReBEC) under the number RBR-5bxtgx (date of registration: November 1, 2019). Participants will be informed about all study procedures, and those who agree will be asked to sign the informed consent form prior to their enrollment in the study.

### Study setting and recruitment

This trial will be conducted in the Laboratory of Research in Women’s Health at UFSCar’s Physical Therapy Department. The study will be disclosed on social media, websites, and leaflets; prospective participants will be invited through an informal invitation and social media. All women who want to take part in the study will have their names, ages, and telephone numbers registered for posterior contact. After the first telephone contact, women will be screened for eligibility criteria, and eligible women will be invited to participate in the study. This study will be carried out following the Standard Protocol Items: Recommendations for Interventional Trials (SPIRIT) statement for clinical trial protocols [30].

### Sample size calculation

A recent systematic review showed that the effect size of TENS for PD was about 1.384 [31]. However, other studies that have used TENS as a primary intervention for PD did not look into the economic costs of the intervention or had other confounding factors analyzed as well. As such, the authors of the present study opted for a more conservative sample size calculation, with a much lower effect size. Therefore, the sample size calculation was performed with G*Power software (v.3.1.9.2, Dusseldorf, Germany), considering the comparison of three independent groups at five timepoints (baseline and months 1, 2, 3, 6 and 12 after baseline), with analysis of variance for repeated measures, a β value of 15%, an α of 5%, and an effect size (f) of 0.20, considering a dropout rate of 15%, resulting in 174 participants in the study (58 participants per group).

### Eligibility criteria

Participants’ inclusion criteria are as follows: women, nullipara, aged between 18 and 45 years, regular menstrual cycle, and presence of PD (pelvic or lower abdominal pain, occurring from 48 h before the onset of the menstrual period to 72 h after the first day of menstruation [2]) with pain intensity greater than or equal to four points in the Numerical Rating Scale (NRS) on the three menstrual cycles prior to their participation. The NRS considers 0 as no pain, 1–3 as mild pain, 4–7 as moderate pain, and 7–10 as severe pain [32], and only women with moderate to severe pain will be included, as more women rate their PD pain as moderate [33]. In addition, moderate to severe menstrual pain is associated with decreased quality of life [34]. Exclusion criteria for this study are as follows: pregnant, women using any kind of intrauterine device, women with skin lesions in the area where the electrodes will be placed, women with self-reported neurological or cardiac diseases, and women diagnosed with gynecological disorders (endometriosis, adenomyosis, uterine fibroids, etc.) that may be associated with secondary dysmenorrhea. The presence of any signs of skin irritation that may be associated with electrostimulation will be considered as a criterion to discontinue treatment. No concomitant care or interventions will be encouraged or forbidden during the study, but rather registered in the participant’s file.

### Assessment of clinical outcomes

Participants who agree to participate will be assessed by a blinded assessor who will check for eligibility criteria. Subsequently, the blinded assessor will collect participants’ demographic and anthropometric data and information regarding menstrual pain medication use. Next, all eligible participants will be randomized and allocated into three groups: active TENS (aTENS), placebo TENS (pTENS), and minimum intervention group (booklet). The same blinded assessor will evaluate primary and secondary outcomes.

The primary outcome is pain intensity at baseline and three and six months after randomization. The secondary outcomes are pain intensity (only at 12 months after randomization, for the cost-utility analysis), health-related quality of life at 6 and 12 months after randomization, and pain intensity at each session before and after treatment for three months for the TENS groups. All assessment sessions are described in Table 1.

**Table 1:** Timeline for the schedule of enrollment, assessments, and interventions.

	Enrollment	Month 1		Month 2		Month 3		Follow-up	
		Baseline	Post	Pre	Post	Pre	Post	Month 6	Month 12
Eligibility criteria	x								
Informed consent	x								
Demographic and anthropometric data	x								
**Primary outcome**									
Pain intensity (NRS)		x					x	x	
**Secondary outcome**									
Pain intensity (NRS)			x	x	x	x			
Health-related quality of life (SF-36 and SF-6D)		x				x		x	x
**Interventions**									
Active TENS			x	x		x			
Placebo TENS			x	x		x			
Educational Booklet			x						

To evaluate pain intensity, an 11-point NRS will be used, where 0 (zero) represents no pain and 10 (ten) represents the greatest imaginable pain. Participants will be instructed to rank their pain according to the appropriate point of the scale [35].

Health-related quality of life will be evaluated for Short-Form 6 dimensions (SF-6D) [36, 37], a multi-attribute utility instrument translated and validated to Portuguese/Brazil [38]. It measures health on six dimensions: physical functioning, role limitations, social functioning, pain, mental health, and vitality. The SF-6D unique score varies from 0 (worst health state) to 1 (best health state) and represents the strength of an individual’s preference for a given health state.

Conventionally, it has been recommended to use SF-6D together with the 36-Item Short Form Health Survey questionnaire (SF-36) [37]. The SF-36 [39, 40] assesses health-related quality of life in eight domains: physical functioning, role physical, bodily pain, general health, vitality, social functioning, role-emotional, and mental health. The SF-36 scores represent a health status and range from 0 (worst health status) to 100 (best health status) for each domain.

The baseline assessment will be performed face-to-face for all participants. Participants allocated to the TENS groups will respond to pain intensity face-to-face, before and after each treatment session, during three consecutive menstrual cycles. During this period, participants allocated to the educational booklet will be assessed using text messages and/or video calls with WhatsApp®. In the three-month assessment, the participants of all groups will also be asked about their satisfaction with the treatment. The 6- and 12-month assessments will be conducted using WhatsApp®. The timeline for the schedule for enrollment, assessment, and interventions is presented in Table 1.

### Economic assessment

Economic assessment will be done from a healthcare and societal perspective. Incremental cost per quality-adjusted life-years (QALYs) of the interventions will be used for cost-effectiveness and cost-utility analysis, considering both healthcare and societal perspectives, measuring direct and indirect costs. The Numerical Rating Scale will be used for cost-effectiveness analysis, and the SF-6D questionnaire will be used for cost-utility analysis. Cost data will be collected by a cost questionnaire that will be sent to the participant via WhatsApp® at 3, 6, and 12 months after baseline. All costs related to menstrual pain will be considered, either direct (costs of the participant to health systems, public and private, and out-of-pocket expenses) and indirect (unpaid productivity loss), which include intervention, healthcare, informal care, and unpaid productivity costs. Intervention costs will be estimated based on the physical therapy board fee schedule (according to the Federal Physical Therapy and Occupational Therapy Council, USD 11.32 per TENS application– 2020 fee which is equivalent to R$ 64.63 rate converted to US dollars using the 04/02/2021 conversation rate [41]). Healthcare costs, including primary and secondary healthcare costs, will be priced using Brazilian standard costs. Both prescribed and over-the-counter medication use will be priced using the prices of the Brazilian pharmaceutical websites and medical services fee schedule.

A Portuguese/Brazil validated version [42] of the Stanford Presenteeism Scale [43] will be used to measure presenteeism from paid work, and the estimated price of productivity losses per sickness absence day for women, according to the average wage in the state of São Paulo per hour.

### Randomization and concealed allocation

Prior to the baseline assessment, participants will be randomized into three groups: aTENS, pTENS, and booklet. Randomization will be generated by a computer program, “Randomization Main,” and performed by a participating researcher not involved with the participants’ recruitment or evaluation. The age range for participants is relatively broad, but both the eligibility criteria and the randomization process help to ensure that groups will be equivalent at the beginning of the experiment, turning them comparable at the end of the experiment [44]. Concealed allocation will be achieved using sequentially numbered, sealed, and opaque envelopes. The envelopes will be stored in a safe cabinet that only the allocation investigator will have access to; the envelopes will not be opened until immediately prior to the first intervention session [45]. The allocation ratio will be 1:1:1.

### Interventions

For the TENS group, two Neurodyn II (IBRAMED ®, Brazil) devices will be used. Before starting the trial, one researcher not involved in the study will assign a number (1 or 2) to each of the two TENS devices: one to be the active TENS, while the other will be programed by IBRAMED ® to be used as a placebo. To achieve and ensure blinding in the pTENS group, the participants will be connected to the TENS unit in the exact same way as participants of the aTENS. The active indicator of the unit emits light and sound just as the active device but will deliver electrical stimulation in the placebo mode, described below. Participants and physical therapists will be blinded throughout the treatment.

**Fig 1:**
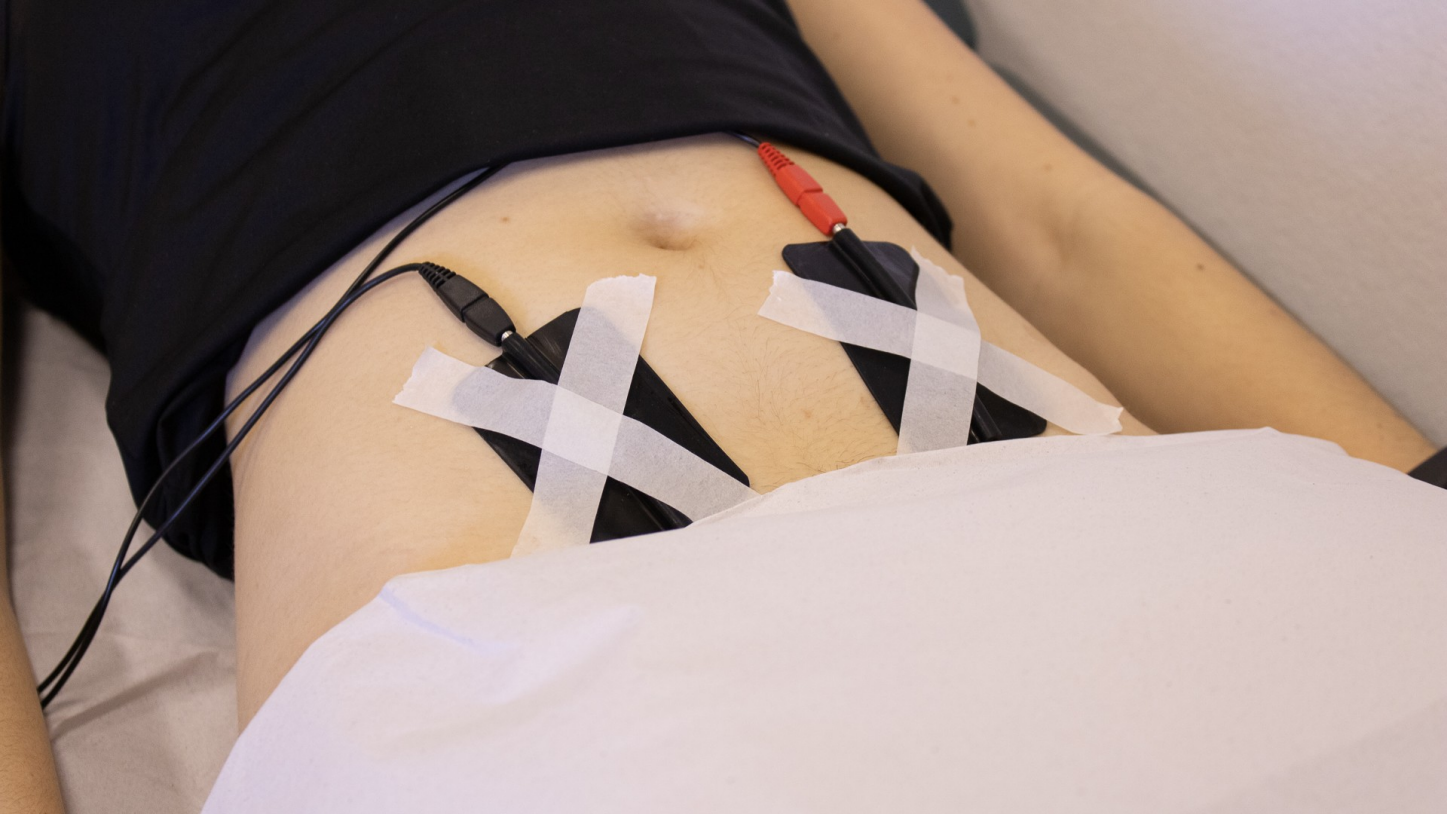


To report the TENS parameters, the recommendations of Barbosa et al. [46] were followed. The protocol was based on Machado et al. [47]: a balanced asymmetric biphasic pulsed will be used in transcutaneous application mode. Two silicone rubber and carbonate electrodes (5X3 cm, Quark®, Brazil) will be coupled with a conductive gel and fixed on both sides of the lower abdomen at the T10-T11 level with micropore tape. The positioning of the electrodes can be observed in Fig 1. The active TENS parameters will be a frequency of 100 Hz, 200 μs pulse duration, and maximal but comfortable current amplitude (sensory level). The current amplitude will be increased until the participant reports feeling the current as a strong tingling sensation [48]. The amplitude will be registered during the entire treatment session. For the placebo TENS, the device will release current with the same parameters (F = 100 Hz, pulse duration of 200 μs) but for only 30 s, and then the current will be ramped off over a 15-s time frame. All participants, including those allocated to the pTENS, will be asked about their sensations during stimulation every 5 min. If a participant in the aTENS perceives a lower sensation, the current amplitude will increase until the sensation is felt again. The current amplitude will not be increased until the motor level, which means that if the motor level is reached, the current amplitude will not be further increased. The pTENS group will also be queried on its intensity every 5 min, and if they ask about the lack of sensation, they will receive an explanation that this is expected on the basis of the sensory habituation that occurs in most people with TENS application [49].

All groups will receive an educational booklet containing information on the anatomy of the pelvis, menstrual pain, and recommendations related to dysmenorrhea. The booklet group will not receive additional treatment and will be advised not to receive treatment elsewhere during the first three months of participation in the study. This educational booklet was validated using a modified Delphi method. A group of experts discussed issues related to the practice of physiotherapy in the treatment of PD, while another group read the identified manuscripts and guidelines related to the topic. The experts had six meetings to discuss the findings and propose the booklet. Subsequently, a group of consultants, including physiotherapists who are specialists in women’s health, further analyzed, made additional contributions, and approved the booklet. Several women with DP were also consulted and approved the final version of the booklet.

### Statistical analysis

Statistical analysis will be performed by a statistician blinded to the allocation of the groups and without involvement in the research. The software SPSS 16.0 and R 3.5.1 will be used for data analysis. All analyses will follow the intention-to-treat principle. The level of significance will be set at 5%. The normality of the residues will be verified by a Shapiro-Wilk test. The mean difference in the effects of the interventions and the difference between the groups will be calculated using linear mixed models with unstructured covariance, which involves the treatment groups, the time (follow-up), and the interaction between the groups over time. All information regarding possible confounding factors (such as body mass index, use of pain medications, etc.) will be recorded and used in the analysis.

To assess the cost-effectiveness of TENS for dysmenorrhea, pain intensity will be used as an outcome, and to perform cost-utility analysis, QALY gained over the treatment period will be used. Mean between-group cost differences will be corrected for their baseline, and incremental cost-effectiveness and cost-utility ratios will be calculated by dividing total costs by the cost differences. The QALY combines the length and quality of life into a single index number between 0 and 1, where 0 corresponds to a health state judged to be equivalent to death and 1 corresponds to optimal health [50]. Subsequently, to compare health costs between groups, we will calculate a deterministic cost-utility value and a probabilistic one using bootstrapping techniques and computing confidence intervals. The 95% confidence intervals will be obtained by bias-corrected and accelerated bootstrapping, choosing 5,000 interactions. Sensitivity analysis will be performed to explore the uncertainty of each parameter by examining the changes in the results in the range of parameter values, using the Kaplan-Meier method and the between-group comparison by the Log-Rank test [51].

## Discussion

### Potential impact and significance of the study

This is the first study that proposes a health economic evaluation alongside assessing the benefits of using TENS to treat PD symptoms. As such, the results of the present study may contribute to the PD research agenda. Although TENS is used to treat PD symptoms [16] and has been shown to reduce pain with minimum adverse effects [15], the studies are considered of low to moderate quality [16] and lack information on parameters and clinical considerations [15], which makes it difficult to compare results and to establish the best parameters of electrical stimulation. Understanding whether TENS is really cost-effective for PD pain treatment opens possibilities to include this resource as a recommendation, and with that, call the attention of both professionals and women to the fact that PD can be treated. Improving PD symptoms may mean, at last, that the economic impact of this disease may be reduced by either decreasing presenteeism or the use of medications and health care services due to PD pain.

### Contribution to professionals and patients

It is not very common for women to seek help due to PD pain, usually because PD is not recognized as a legitimate health issue, not only by women, but also by health care providers and society [52]. The present study can attract attention to the issue and, as such, allow health care providers and women to start looking at PD as a health issue that has nonpharmacological interventions as a treatment resource. If TENS is proven cost-effective for the treatment of PD pain, health care managers may recommend the use of electrical stimulation for this common symptom, and some professionals, such as physical therapists and nurses, can start assisting women during this period, practicing evidence-based care. Women will have more information on the possible nonpharmacological interventions to treat their pain, with more resources at their disposal, and have their quality of life improved. This may lead to an increase in the seek for health care related to the menstrual period, but also to a decrease in the number of working hours or days lost due to PD symptoms.

### Strengths and weaknesses of the study

The strengths of the present study are that this is the first prospective study that aimed to assess the cost-effectiveness of TENS for PD pain. This is a randomized controlled clinical trial in which researchers, participants, and physical therapists are blinded to participants’ allocation and to the aim of the present study. The sample size was appropriately calculated to provide enough power to detect differences in the primary outcome. The statistical plan analysis includes the intention-to-treat analysis and intends to assess all women who discontinue intervention. One limitation of the present study is that even though participants allocated to the TENS groups (both active and placebo) will be blinded to their allocation, it is not possible to blind participants in the Booklet group.

### Future research

The present study opens new possibilities for new investigations on nonpharmacological interventions for PD. Future studies could include combinations of TENS and more long-term interventions such as exercises, yoga, and manual therapy, and analyze the cost-effectiveness of the combination of these techniques.

## Supporting information

S1 Checklist SPIRIT 2013 checklist: Recommended items to address in a clinical trial protocol and related documents*. (DOC)

S1 File (PDF)

S2 File (PDF)
