# Supplementary material for: Transcutaneous electrical nerve stimulation for women with primary dysmenorrhea: Study protocol for a randomized controlled clinical trial with economic evaluation
Source: PLoS One. 2021 May 20;16(5):e0250111. doi: 10.1371/journal.pone.0250111 (PMC8136645; doi:10.1371/journal.pone.0250111)
Supplement: S1 File — (PDF) [file pone.0250111.s002.pdf]

PARECER CONSUBSTANCIADO DO CEP

DADOS DO PROJETO DE PESQUISA

Título da Pesquisa: ESTIMULAÇÃO ELÉTRICA NERVOSA TRANSCUTÂNEA EM MULHERES COM DISMENORREIA PRIMÁRIA: ESTUDO CLÍNICO RANDOMIZADO COM AVALIAÇÃO ECONÔMICA

Pesquisador: Patricia Driusso

Área Temática:

Versão: 3

CAAE: 16530619.3.0000.5504

Instituição Proponente: Programa de Pós-Graduação em Fisioterapia - PPGFt

Patrocinador Principal: Financiamento Próprio

DADOS DO PARECER

Número do Parecer: 3.588.121

Apresentação do Projeto:

Trata-se de um estudo clínico, randomizado com avaliação econômica, que busca avaliar em mulheres que apresentam dismenorreia primária os efeitos da utilização da Estimulação Elétrica Nervosa Transcutânea (TENS)sobre a intensidade da dor em mulheres acometidas pela DP e estimar o custo-efetividade e custoutilidade da TENS para mulheres com DP. As participantes serão mulheres com idade superior a 18 anos, nulíparas, com ciclo menstrual regular e diagnóstico de DP, possuindo relato de dor igual ou maior a quatro pontos na Escala Numérica da Dor (END). As participantes serão avaliadas por meio de dados sociodemográficos, clínicos e econômicos e, também, pela aplicação dos instrumentos: Questionário Genérico de Avaliação de Qualidade de Vida (SF-36) e Questionário Genérico de Avaliação de Qualidade de Vida 6 Dimensões (SF-6D). As participantes serão aleatorizadas e alocadas em três grupos: Grupo controle (TENS placebo), Grupo intervenção (TENS) com frequência de 100Hz e duração de pulso de 200s e Grupo intervenção mínima (cartilha). As participantes alocadas no grupo controle (TENS placebo) e no grupo intervenção (TENS) serão submetidas ao tratamento com TENS placebo e TENS ativo, respectivamente. A aplicação da TENS será realizada dois dias antes até três
dias depois do primeiro dia da menstruação, quando a participante referir dor, durante três ciclos menstruais consecutivos, e as participantes também serão acompanhadas por mais três ciclos

Endereço: WASHINGTON LUIZ KM 235
Bairro: JARDIM GUANABARA **CEP:** 13.565-905
UF: SP **Município:** SAO CARLOS
Telefone: (16)3351-9685 **E-mail:** cephumanos@ufscar.br

consecutivos.

Objetivo da Pesquisa:

Objetivo Primário: Avaliar o efeito da aplicação da TENS sobre a intensidade da dor em mulheres acometidas pela DP.
Objetivo Secundário: Avaliar o custo efetividade e custo utilidade da TENS em mulheres com DP

Avaliação dos Riscos e Benefícios:

Riscos: algumas perguntas podem remeter a algum desconforto, evocar sentimentos ou lembranças desagradáveis. Também podem ocorrer sinais de irritação da pele devido o uso da TENS, como vermelhidão embaixo ou em torno dos eletrodos. Se houver isso acontecer, os responsáveis pela pesquisa irão te auxiliar imediatamente com compressa de água Benefícios: tratamento para a cólica menstrual e conhecer melhor os aspectos relacionados à sua qualidade de vida

Comentários e Considerações sobre a Pesquisa:

Pesquisa importante para área. Cronograma apresentado está adequado. O recrutamento das participantes do estudo será realizado por meio de anúncios em panfletos, mídias e redes sociais na cidade de São Carlos – SP (Apêndice II). Tal comunicação só será divulgada após a aprovação do projeto pelo Comitê de Ética em Pesquisa da Universidade Federal de São Carlos (UFSCar). O estudo ocorrerá no Laboratório de Saúde da Mulher do Departamento de Fisioterapia da Universidade Federal de São Carlos.

Considerações sobre os Termos de apresentação obrigatória:

Folha de rosto apresentada, assinada pelo pesquisador responsável e pela diretoria de centro. Prevê um número de participantes de 174.
TCLE apresentado, está adequado. Pesquisadora inlcuiu, conforme solicitação no parecer anterior a assistência integral e garantia de indenização caso ocorra algum dano decorrente da pesquisa

Conclusões ou Pendências e Lista de Inadequações:

Projeto adequado após adequações solicitadas no parecer anterior.
Aprovado

Considerações Finais a critério do CEP:

Este parecer foi elaborado baseado nos documentos abaixo relacionados:

Tipo Documento	Arquivo	Postagem	Autor	Situação
Informações Básicas do Projeto	PB_INFORMAÇÕES_BÁSICAS_DO_PROJETO_1383758.pdf	16/09/2019 13:15:32		Aceito
TCLE / Termos de Assentimento / Justificativa de Ausência	TCLE3.pdf	16/09/2019 13:15:24	Patricia Driusso	Aceito
Projeto Detalhado / Brochura Investigador	Projeto.pdf	29/06/2019 09:12:03	Patricia Driusso	Aceito
Folha de Rosto	FRDismenorreia.pdf	29/06/2019 09:06:48	Patricia Driusso	Aceito

Situação do Parecer:
Aprovado

Necessita Apreciação da CONEP:
Não

SAO CARLOS, 20 de Setembro de 2019

Assinado por:
Priscilla Hortense
(Coordenador(a))

Endereço: WASHINGTON LUIZ KM 235
Bairro: JARDIM GUANABARA **CEP:** 13.565-905
UF: SP **Município:** SAO CARLOS
Telefone: (16)3351-9685 **E-mail:** cephumanos@ufscar.br
