# Supplementary material for: Transcutaneous electrical nerve stimulation for women with primary dysmenorrhea: Study protocol for a randomized controlled clinical trial with economic evaluation
Source: PLoS One. 2021 May 20;16(5):e0250111. doi: 10.1371/journal.pone.0250111 (PMC8136645; doi:10.1371/journal.pone.0250111)
Supplement: S2 File — (PDF) [file pone.0250111.s003.pdf]

Translation – Ethics Committee Register

UFSCAR – UNIVERSIDADE FEDERAL DE SÃO CARLOS

Ethics Committee Sentence upon Research Project

RESEARCH PROJECT DATA

Research title: Transcutaneous electrical nerve stimulation in women with primary dysmenorrhea: randomized controlled trial with economic evaluation.

Researcher: Patricia Driusso

Theme area:

Version: 3

CAAE (Presentation Certificate of Ethics Assessment): 16530619.3.0000.5504

Proponent Institution: Physical Therapy Post-Graduate Program (PPGFt)

Main Sponsor: Own financing

SENTENCE DATA

Sentence Number: 3.588.121

Project Presentation:

The present research is a randomized clinical trial with economic evaluation that aims to assess in women that present primary dysmenorrhea (PD) the effects of using Transcutaneous Electrical Nerve Stimulation (TENS) on pain intensity in women with PD. Participants will be women aged over 18 years old, nulliparous, with regular menstrual cycle and PD diagnostics, with a pain report equal or greater than four points in the numerical rating scale (NRS). Participants will be assessed regarding sociodemographic, clinical and economic data, and also, by the use of the instruments: Medical Outcomes Study 36-Item Short Form Health Survey (SF-36) and the Generic Quality of life assessment in 6 dimensions (SF-6D). Participants will be randomized and allocated in three groups: control group (placebo TENS), intervention group (TENS) with frequency of 100Hz and pulse duration of 200s and minimal intervention group (booklet). Participants allocated in the control (TENS placebo) and intervention (TENS) groups will undergo treatment with placebo TENS and active TENS, respectively. TENS application will be performed two days before until three days after the first menstruation day, when participant reports pain, during three consecutive menstrual cycles, and participants will also be followed up during three other consecutive cycles.

Research Aims:

Primary objective: assess the effects of TENS application on pain intensity in women who suffer from PD.

Secondary objective: assess cost-effectiveness and cost-utility of TENS on women with PD.

Risks and benefits assessment:

Risks: some questions may refer to some discomfort or evoke unpleasant feelings and memories. Signs of skin rash may also occur due to TENS use, such as reddishness under or around electrodes. If this happens, those responsible for the research will immediately assist with water compresses. Benefits: treatment for menstrual pain and to know better aspect related to quality of life.

Comments and Considerations on the Research:

Important research on the field. Presented schedule is adequate. Participants recruitment will be performed by announces on leaflets, conventional and social media in the city of São Carlos – SP (Appendix II). Such communication will only be disclosed after Federal University's Research Ethics Committee approval. The study will occur on the Laboratory of Research on Women's Health of the Physical Therapy Department of the Federal University of São Carlos.

Considerations of the Terms of mandatory presentation:

Cover sheet presented is signed by the main researcher and the center board. The cover sheet predicts a number of 174 participants.

Consent Form (TCLE) presented is adequate. Researcher has included, as asked in the previous sentence the integral assistance and the guarantee of compensation in case any damage occurs due to the research.

Conclusions or pendency and list of unsatisfactory issues:

Project adequate after changes asked for in the previous sentence.

Approved

Final considerations at the discretion of the Ethics Committee:

This sentence was elaborated based on the documents listed below (*this is the list of documents presented to the Ethics Committee*):

Tipo Documento	Arquivo	Postagem	Autor	Situação
Informações Básicas do Projeto	PB_INFORMAÇÕES_BÁSICAS_DO_PROJETO_1383758.pdf	16/09/2019 13:15:32		Aceito
TCLE / Termos de Assentimento / Justificativa de Ausência	TCLE3.pdf	16/09/2019 13:15:24	Patricia Driusso	Aceito
Projeto Detalhado / Brochura Investigador	Projeto.pdf	29/06/2019 09:12:03	Patricia Driusso	Aceito
Folha de Rosto	FRDismenorreia.pdf	29/06/2019 09:06:48	Patricia Driusso	Aceito

Sentence situation:

Approved

Need of National Ethics Committee Approval:

No

São Carlos, September 20th 2019

____

Signed by:

Priscilla Hortense

(Coordinator)
